# Biosensor-Based Platforms for the Detection and Screening of *Mycobacterium leprae* Infection

**DOI:** 10.1021/acsinfecdis.5c00851

**Published:** 2026-01-21

**Authors:** Augusto César Parreiras de Jesus, Ana Laura Grossi de Oliveira, Flavia Di Scala, Cristiane Alves da Silva Menezes, Lilian Lacerda Bueno, Bart van Grinsven, Rocio Arreguin-Campos, Ricardo Toshio Fujiwara, Thomas J. Cleij

**Affiliations:** † Sensor Engineering Department, Faculty of Science and Engineering, 5211 Maastricht University, Duboisdomein 30, 6200MD Maastricht, The Netherlands; ‡ Post-Graduate Program in Infectious Diseases and Tropical Medicine, School of Medicine, Federal University of Minas Gerais, Av. Prof. Alfredo Balena 190, 30130-100 Belo Horizonte, Brazil; § Department of Clinical and Toxicological Analysis, Faculty of Pharmacy, 28114 Federal University of Minas Gerais, Av. Pres. Antônio Carlos 6627, 31270-901 Belo Horizonte, Brazil; ∥ Department of Parasitology, Institute of Biological Sciences, Federal University of Minas Gerais, Av. Pres. Antônio Carlos 6627, 31270-901 Belo Horizonte, Brazil

**Keywords:** leprosy, Mycobacterium leprae, biosensors, point-of-care systems, electrochemical techniques, neglected tropical diseases, diagnostic techniques and procedures

## Abstract

Leprosy remains an important neglected tropical disease with about 200,000 new cases detected annually worldwide. Although the disease is highly responsive to treatment, a timely and accurate diagnosis continues to be a critical barrier to disease control. Traditional diagnostic methods, including PCR, bacilloscopy, histopathology, and serology, are hindered by limited sensitivity, procedural complexity, and restricted accessibility in resource-constrained settings. This review summarizes studies from the past decade on biosensor-based strategies for leprosy diagnosis. Biosensor platforms for leprosy include electrochemical, piezoelectric, and optical systems, with recent innovations encompassing immunosensors, biomimetic, and DNA-based approaches, some achieving diagnostic accuracies above 90%. These platforms employ different bioreceptors such as conjugated peptides, DNA probes, and molecularly imprinted polymers. Certain platforms can also differentiate paucibacillary from multibacillary cases, addressing a critical limitation of the current methods. These capabilities highlight the potential of biosensors as powerful tools for point-of-care testing. However, clinical translation is constrained by challenges such as affordability, robustness under field conditions, and the lack of large-scale validation studies. Additional operational barriers, including regulatory approval, supply chain logistics, and user training, must also be addressed. Future progress will depend on multidisciplinary strategies, integrating novel biomarker discovery as recognition elements and exploring detection systems previously used for other mycobacterial and infectious diseases. Large multicenter trials and user-centered design approaches are essential for clinical implementation. By overcoming these challenges, biosensors have the potential to redefine leprosy diagnostics, enabling earlier detection and improved surveillance, and accelerating progress toward global elimination goals.

## Graphical abstract

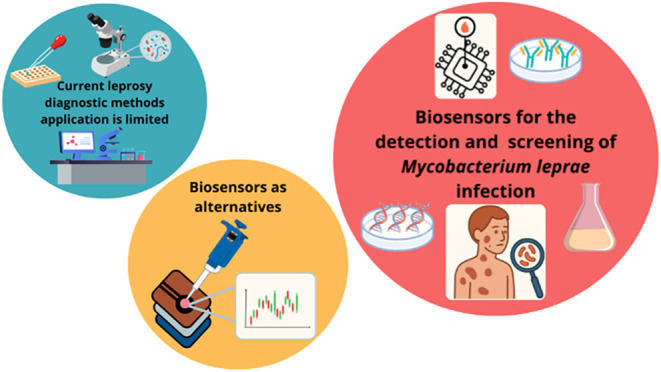

## Introduction

Leprosy is one of the neglected tropical diseases with a persistent worldwide public health impact. This ancient infectious disease, caused by *Mycobacterium leprae*, damages the peripheral nerves and subsequently affects the skin, ultimately leading to severe physical disabilities if left untreated.^1,2^ Despite global efforts to eliminate the disease, around 200,000 new cases are reported annually, with the majority occurring in India, Brazil, and Indonesia.^3^ Early and accurate diagnosis, followed by prompt treatment initiation, is essential to prevent irreversible nerve damage and interrupt bacterial transmission.^4,5^

Although leprosy is highly responsive to treatment, its diagnosis remains a major challenge, particularly in low- and middle-income countries where the disease burden is greatest.^6,7^ As clinical manifestations primarily drive case detection, diagnosis is frequently established only after symptoms have already appeared.^5^ In the 1980s, the World Health Organization (WHO) introduced a simplified classification based on the bacillary index (BI), categorizing cases into paucibacillary (PB) and multibacillary (MB) forms. Patients classified as PB present negative bacilloscopy, fewer than five skin lesions, and/or involvement of a single nerve trunk, whereas MB patients present positive bacilloscopy, more than five skin lesions, and/or involvement of more than one nerve trunk.^8^

Early detection remains essential to preventing the progression of cellular damage. From a laboratory perspective, major diagnostic challenges persist, as *M. leprae* is a fastidious organism that cannot be cultured *in vitro*.^9,10^ Consequently, several conventional laboratory techniques, such as bacilloscopy, histopathology, and serology, have been implemented to complement the clinical evaluation. However, these methods are limited by suboptimal sensitivity in PB cases, invasive sample collection procedures, and the need for specialized personnel, which brings urgent demand for more accurate, accessible, and file-adapted diagnostic tools.^11^

In addition to technical limitations, the consequences of inadequate or delayed diagnosis extend far beyond clinical outcomes, profoundly affecting patients’ social and economic well-being. Improved diagnostic tools have the potential to significantly mitigate these consequences caused by delayed or missed leprosy diagnoses. The social and economic impacts of untreated leprosy and other neglected diseases are substantial, leading to permanent disability, social rejection, stigma, and household costs estimated at 33 billion dollars globally.^12^ Quantitative assessments show that leprosy late case detection results in long-term disability, reduced work capacity, and substantial indirect costs to affected households, particularly in low-income settings.^13,14^ In China, for example, the mean annual cost per patient including transportation, supplementary medications, and income loss represented up to 38% of family income.^14^ Similarly, in Ghana, despite free multidrug therapy, around 60% of families experienced catastrophic health expenditures due to leprosy-related disabilities.^13^ This highlights the indirect costs associated with the disease.

Cost-effectiveness analyses demonstrate that novel diagnostic algorithms, by enabling earlier detection and timely treatment, can substantially reduce disability adjusted life years (DALYs) and the overall economic burden, yielding an incremental cost-effectiveness ratio of USD 616.46 per undiagnosed leprosy case avoided.^15,16^ In Cameroon, for instance, leprosy was found to reduce the number of working days by 115 days.^17^ Moreover, socioeconomic modeling in Brazil and other endemic countries indicates that the poorest segments of the population are at higher risk, so reducing diagnostic delay not only prevents disabilities but also improves labor productivity and helps break the cycle of poverty.^18,19^ Therefore, developing and implementing more accurate and accessible diagnostic tests are crucial to alleviate the broader social and economic impacts of leprosy, ultimately contributing to poverty reduction and sustainable disease control.

Herein, this review examines the past decade of research on novel biosensor technologies for the rapid and reliable detection of leprosy, emphasizing advances that could enable more sensitive, accessible, and field-applicable diagnostic approaches. The strategy used for the literature search is described in the Supporting Information. We also draw insights from biosensing strategies applied to other *Mycobacterium*-related diseases, which may shed light on the development of leprosy-specific platforms. Before exploring these emerging technologies, we provide a concise overview of the current laboratory methods supporting leprosy diagnosis, setting the stage for the discussion of future directions.

### Current Leprosy Diagnostic Methods

Leprosy diagnosis remains mainly clinical, as no laboratory method has yet achieved gold standard status.^20,21^ Complementary tools, including PCR, bacilloscopy, histopathology, and serology, are briefly discussed below. Historically, the Mitsuda skin test was widely employed.^22^ However, its current application has been limited to research settings.^23–25^ Other techniques, such as electrophysiological tests, ultrasonography, and magnetic resonance neurography, help assess neural impairment^25^ but are beyond the scope of this review.

#### Polymerase Chain Reaction (PCR)

Polymerase chain reaction (PCR) is currently regarded as the most reliable laboratory method for confirming leprosy with true positive results. Nevertheless, PCR remains too expensive for routine use in low-resource settings, requires trained personnel, and is generally restricted to major urban centers.^11,26^ The most commonly identified sequences are the *M. leprae* repetitive element (RLEP), *M. leprae* sodA mRNA, 16S rRNA, and the *M. leprae* Pra gene.^27–31^ The summary sensitivity of conventional PCR was approximately 75% with a specificity of 94.5%. For qPCR, the sensitivity was around 79% and the specificity was about 89%.^32^ The molecular detection process (Figure 1) begins with the collection of a skin sample, followed by the extraction of *M. leprae* DNA, amplification through temperature cycles, and detection by gel electrophoresis (for conventional PCR) or fluorescence (for qPCR). A positive result confirms infection, and the typical turnaround time is approximately 1–3 days.

**Figure 1:**
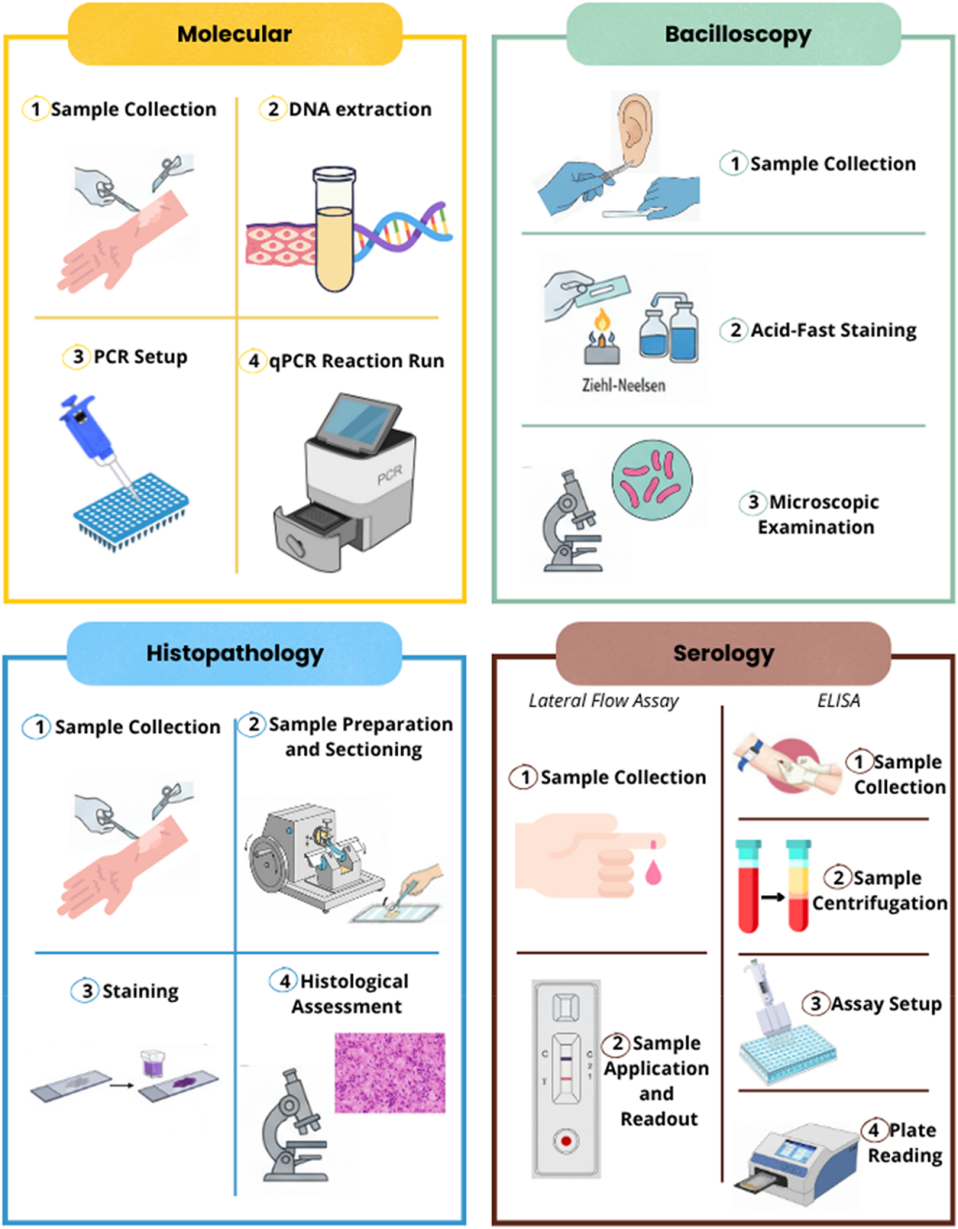
Overview of the laboratory workflows currently used for the diagnosis and classification of leprosy. The figure summarizes the key steps involved in the four main laboratory approaches: molecular testing, bacilloscopy, histopathology, and serology.

#### Bacilloscopy

Slit-skin smear (SSS) microscopy involves the collection of material from active lesions or areas with altered sensitivity, as well as from the earlobes and the contralateral elbow, which are sites known to harbor high bacillary loads in untreated patients.^33^ The samples are stained using the Ziehl–Neelsen method and examined under a microscope. Results are expressed as BI based on a logarithmic scale ranging from 0 (no bacilli in 100 fields) to 6 (more than 1000 bacilli per field).^34,35^ This method is highly specific but exhibits low sensitivity, particularly in PB cases, due to its high detection threshold. Overall, SSS shows specificity between 91 and 100% and sensitivity ranging from 31 to 78%.^36–38^ Accurate bacilloscopy requires well-trained personnel, proper infrastructure, and standardized procedures, as slide interpretation is operator-dependent and subject to variability. The procedure involves collecting material from skin lesions, earlobes, or other sites with altered sensitivity, followed by fixation, staining, and microscopic examination to identify acid-fast bacilli (AFB) (Figure 1). A positive result confirms the presence of the bacteria, and the typical turnaround time is a few hours, often on the same day, depending on the laboratory workflow.

#### Histopathology

Histopathological findings may also support leprosy diagnosis and classification.^39^ Biopsies are typically obtained from the margins of the most recent and active lesions, and stained with hematoxylin–eosin and Fite–Faraco stains to evaluate cellular infiltrates, granulomas, and bacilli (Figure 1).^40^ The typical turnaround time for complete procession is 24–48 h, although it may take longer in some laboratories.^41^ Although both slit-skin smear microscopy and histopathology exhibit high specificity, their sensitivity is limited, particularly in PB cases.^42,43^ It is estimated that the diagnostic specificity of histopathology and skin biopsies ranges from 70 to 72%, with sensitivity exhibiting notable variability, ranging from 49 to 70%.^44,45^

#### Serological Tests: Cellular and Humoral Responses

Among serological tests, the phenolic glycolipid-I (PGL-I) antigen has been the most extensively studied. PGL-I is a species-specific glycolipid located on the *M. leprae* cell wall, providing immunological specificity for antibody detection assays. While these tests are useful for detecting MB patients, they show limited sensitivity in PB cases due to low systemic antibody levels.^46^ Additional antigens explored in serological studies include NDO-HSA (natural octyl disaccharide bound to human serum albumin), LID-1 (the fusion protein product of the ML0405 and ML2331 genes), and NDO-LID (a combination of LID-1 and NDO). However, none have demonstrated sufficient performance for PB detection. Serological tests have demonstrated sensitivity ranging from 80 to 100% in MB cases, whereas in PB cases the sensitivity is considerably lower, ranging from 30 to 60%.^47–50^ Overall, among the available serological assays, the ELISA test showed an approximate sensitivity of 64% and a specificity of 91%. The lateral flow test (LFA) demonstrated a sensitivity of about 68% and a specificity close to 87%.^32^ For serological testing, the procedure began with a blood collection. In ELISA, serum is separated and incubated in antigen-coated plates, followed by the addition of an enzyme-linked secondary antibody and substrate, with color development measured to indicate the antibody presence. The assay can typically be completed in 3–4 h after sample preparation. In lateral flow assays (LFA), blood is applied to a test strip, where antibodies bind to antigens as the sample migrates by capillarity, producing visible test and control lines (Figure 1). Results are generally available within 10–20 min.^51^

According to a study by Costa and colleagues,^15^ the estimated costs (in USD) of different diagnostic tests for leprosy are as follows: slit-skin smear (SSS) microscopy, $2.35; rapid serological tests (RT), $4.80; and PCR (RT-PCR or qPCR), $26.20. In comparison, the Brazilian Ministry of Health is allocating approximately $0.78 for SSS, $13.58 for histopathology, and $5.00 for LFA.^52–54^

Table 1 summarizes the main benefits and limitations of commonly used laboratory techniques to detect *Mycobacterium leprae* infection.

**Table 1:** Comparative Characteristics of Current Laboratory Methods Supporting Leprosy Diagnosis

method	advantages	disadvantages	sample type	accuracy (%)	cost (USD)
PCR	highest sensitivity; robust confirmatory test	high cost, technical complexity, limited accessibility in endemic regions	skin or nerve biopsy	Sen: 75–79	$26.20
				Spe: 89–95	
bacilloscopy	high specificity; widely established method	low sensitivity in PB cases; requires invasive sample collection	dermal smear	Sen: 31–78	$0.78
				Spe: 91–100	
histopathology	provides detailed tissue analysis; high specificity	low sensitivity in PB cases; invasive; requires specialized personnel	skin biopsy	Sen: 49–70	$13.58
				Spe: 70–72	
serology	good performance in MB patients; minimally invasive	inadequate sensitivity for PB detection; not validated for contact screening	serum	Sen: 64–73	$4.80
				Spe: 90–91	

### Biosensors as Alternatives

In recent years, biosensors have emerged as a promising alternative for the rapid, sensitive, and specific detection of infectious diseases. Biosensors are analytical platforms capable of detecting specific biochemical reactions or molecular interactions, such as antigen–antibody recognition, DNA hybridization, or enzymatic activity, and converting them into a measurable signal proportional to the analyte concentration through a transducer.^55,56^ Compared to conventional diagnostic methods, biosensors provide several advantages, including portability, suitability for point-of-care applications, equal or higher sensitivity, and reduced procedural complexity by eliminating steps such as amplification and washing.^11,57^

Several studies indicate that the global biosensor market is experiencing substantial expansion and is emerging as a transformative component within the healthcare sector.^58–61^ According to Grand View Research, the global market for biosensors across all applications was valued at approximately USD 30.0 billion in 2024 and is projected to reach USD 48.6 billion by 2030, corresponding to a compound annual growth rate (CAGR) of 8.6%.^62^ Specifically, the market for biosensors targeting infectious diseases was estimated at USD 543.6 million in 2024, with a projected growth to USD 845.0 million by 2030, reflecting a CAGR of 7.8%, highlighting its potential influence on future healthcare innovations. The broader diagnostic market for neglected tropical diseases, including conventional laboratory tests, was valued at around USD 6.84 billion in 2024 and is expected to reach USD 9.59 billion by 2030, corresponding to a CAGR of 6.0%.^63^

Figure 2 illustrates a planar biosensor configuration. It comprises several key components: (i) a biological receptor exhibiting high sensitivity and selectivity toward the target analyte; (ii) an interface (linking layer) in a liquid environment, chemically functionalized to facilitate the binding of the target to a solid substrate while maintaining its biological activity; (iii) a transducer responsible for converting the biological signal into quantifiable electrical output; (iv) a microelectronics unit, including the detection system and the analogue front-end; and (v) a data-processing unit with a digital readout that ensures noise reduction, amplification, and elaboration of the signal.^64^

**Figure 2:**
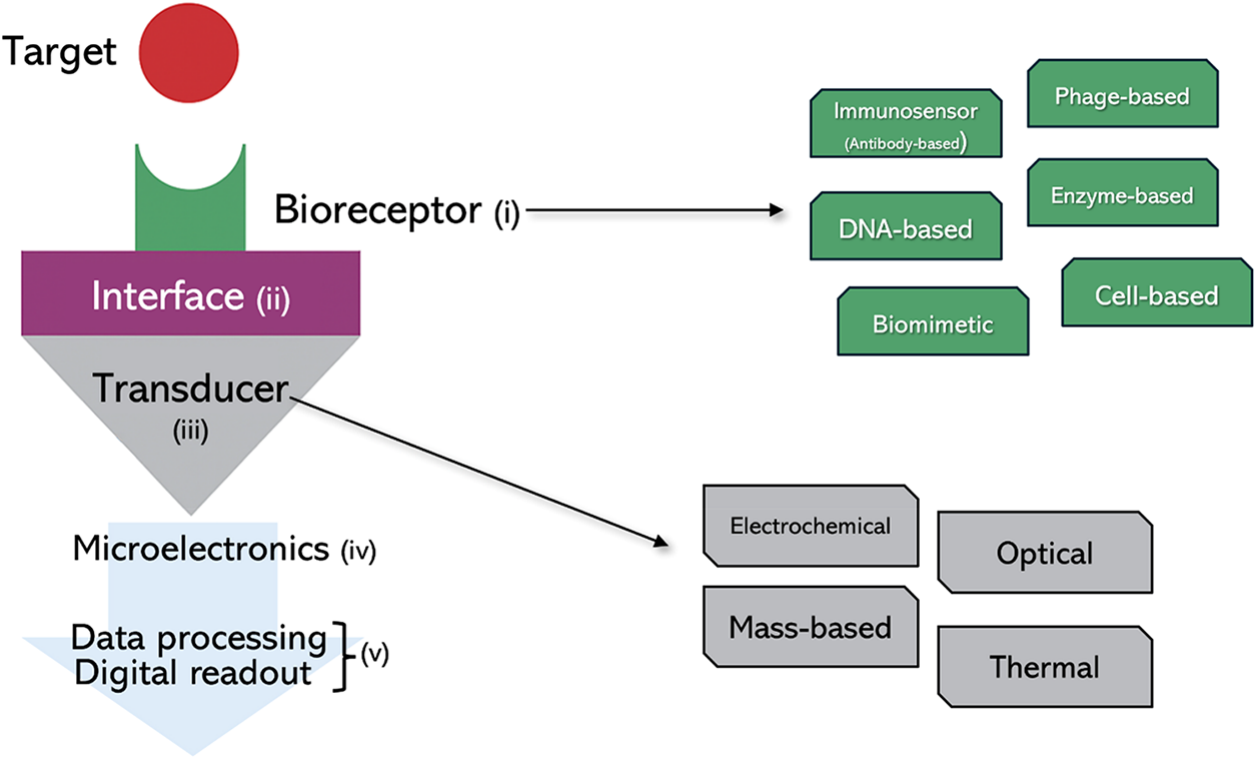
Planar configuration of a biosensor and classification of biosensors. Biosensors can be categorized based on the type of transducer (electrochemical, mass-based, optical, thermal) or the type of bioreceptor (immunosensors, DNA-based, biomimetic, cell-based, phage-based, enzyme-based).

## Biosensors for Leprosy

Biosensors have been successfully employed in the diagnosis of several infectious diseases, including leishmaniasis,^65–67^ schistosomiasis,^68^ malaria,^69,70^ Chagas disease,^71,72^ tuberculosis,^73–75^ Zika,^76^ and dengue.^77^ Unlike traditional diagnostics, biosensors may reduce the interval between sample collection and diagnosis from several days to just minutes or hours.^78,79^ Furthermore, many biosensor platforms operate with minimal laboratory infrastructure, making them suitable for deployment in the field and low-resource environments.^61,80,81^ These features could substantially enhance case detection, allow earlier treatment initiation, and contribute to breaking the transmission chain of leprosy. This is particularly relevant for highly affected countries such as India, Brazil, and Indonesia, where health services are often limited outside major urban centers. The integration of biosensors with potentiometric interfaces, which enhance automation, connectivity, and real-time analysis, further highlights their potential for future diagnostic claims. Such technological progress could accelerate the global leprosy elimination agenda.^4^

Biosensors can be classified based on the type of transducer employed for signal detection, including electrochemical, mass-based, optical, and thermal systems.^82^ Another possible classification is based on the type of bioreceptor, such as immunosensors (antibody-based), DNA-based sensors, biomimetic, cell-based, phage-based, and enzyme-based sensors.^83^ A single biosensor may incorporate more than one type of transducer or bioreceptor. Both classifications, by transducer and by bioreceptor, are illustrated in Figure 2. In the last ten years, several innovative biosensor-based approaches have been proposed for leprosy. Among these, electrochemical transducers have been the most widely applied (Table 2). In 2024, the electrochemical segment accounted for the largest share of the market, comprising 71.7%.^62^ This predominance is attributed to its low detection limits, such as high accuracy and strong specificity.^84^

**Table 2:** Biosensors Developed for Leprosy Diagnosis in the Last Decade and Their Characteristics

study	biosensor type (transducer/receptor)	sample type	detection platform	detection limit/sensitivity	key findings
Almeida et al.^95^	electrochemical/immunosensor	serum	disposable microfluidic device with magnetic particles	sensitivity: 91.2%, specificity: 93.3%, AUC: 0.990	distinguished paucibacillary and multibacillary cases; portable, integrated format
Afonso et al.^100^	electrochemical/DNA-based	biopsy/blood	graphite electrode + poly(4-aminophenol) + methylene blue	1 × 10^–10^ mol/L	high selectivity for DNA target; hybridization signal with methylene blue
Lima et al.^107^	electrochemical-optical/immunosensor	serum	surface plasmon resonance (SPR)	binding affinity to serum antibodies is almost 30-fold lower than ELISA	all leprosy clinical forms were distinguished from controls; household contacts did not present significant SPR angle variation
Kushwaha et al.^115^	electrochemical-piezoelectric/biomimetic (MIP)	blood	electrochemical QCM (EQCM)	0.161 nM	detected low levels of bacterial epitope in blood; high sensitivity and specificity
Yotsumoto Neto et al.^78^	photoelectrochemical/immunosensor	serum	CdS/Ni(OH)_2_/FTO electrodes	reactive up to 1:10240 dilution	detected specific antibodies at high serum dilutions; negatives up to 1:640 showed no response
de Santana et al.^79^	piezoelectric/immunosensor	serum	quartz crystal microbalance (QCM)	detected in 91.7% of patients (ELISA format)	irreversible antibody binding to mimotope; effective in paucibacillary cases; ELISA with amplification improved performance

The electrochemical detection principle relies on the interaction between a biased metal (*working electrode*) and the electrolyte, producing a potential drop across the interface. This process induces charge redistribution and the formation of a double layer, potentially involving electron transfer reactions (redox).^85^ A flat electrode immersed in an electrolyte can be modeled by considering the series between the solution resistance (Rsol) and capacitance (Cdl).^86^ Additionally, ion redistribution at the electrode surface must be accounted for. When the electrode is charged, oppositely charged ions are attracted to form the Inner Helmholtz Plane (IHP), where they become immobilized. Beyond the IHP, solvated ions constitute the Outer Helmholtz Plane (OHP), held by electrostatic forces.^87^ The diffuse layer follows where ions gradually transition from the OHP to the bulk solution. To account for these interfacial phenomena and electrode surface roughness, the constant phase element (CPE) is incorporated into the model.^88^ When sufficient voltage is applied at the metal–liquid boundary, a redox reaction is triggered.^89^ This process is characterized by the charge transfer resistance (Rcell), reflecting the electron transfer barrier, and the Warburg impedance (Z), representing diffusional delays of molecules reaching the reactive surface.^90^ These interfacial phenomena and associated circuit elements, including Rsol, Cdl, CPE, Rcell, and Warburg impedances, collectively determine the baseline impedance of the electrode. The binding of target pathogens to bioreceptors immobilized on the electrode surface alters the local physicochemical properties of this interface. These events modify parameters such as Rcell or CPE components, leading to measurable shifts in the interfacial impedance that constitute the diagnostic signal detected by electrochemical instruments (Figure 3).^91,92^ A variety of these instruments, including cyclic voltammetry, amperometry, impedance spectroscopy, and chronoamperometry, can be employed to track changes in the signal (Icell), resulting from analyte binding upon application of a stimulus (Vcell).^93^

**Figure 3:**
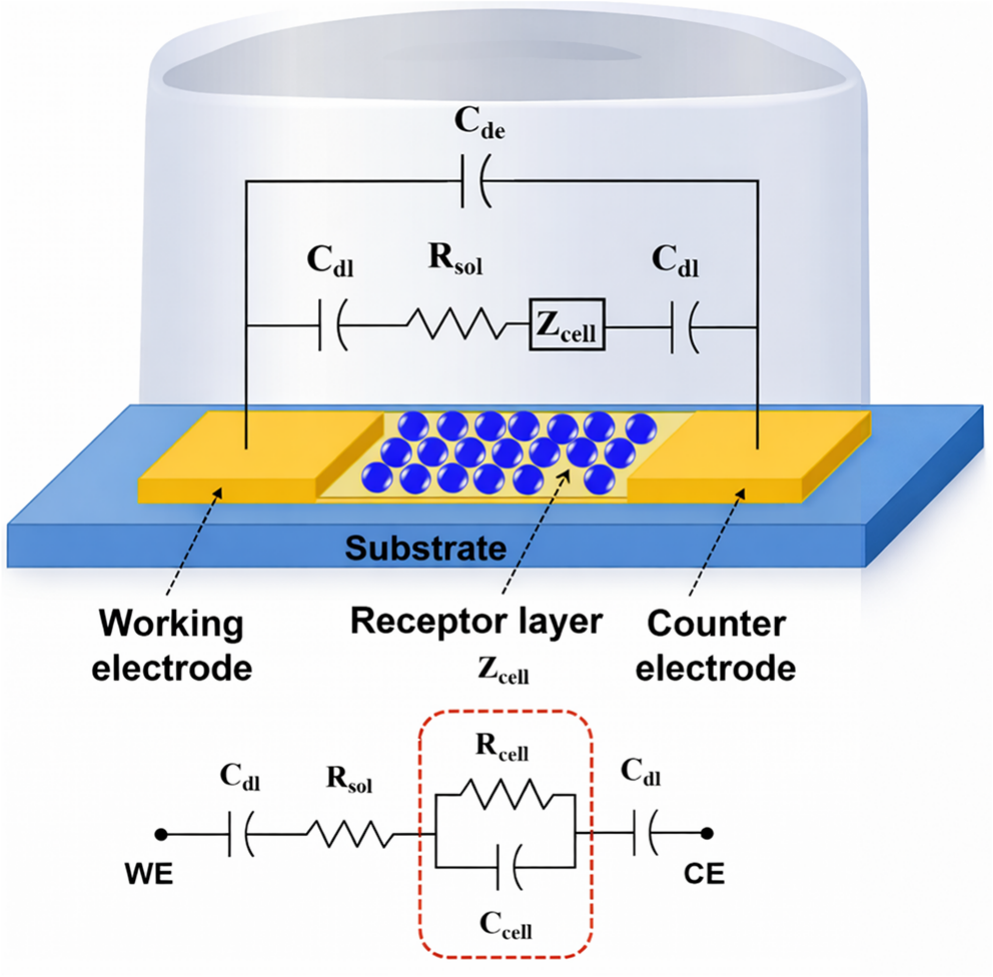
Schematic diagram of the equivalent circuit of an electrochemical biosensor, where Cdl represents the electrical double-layer capacitance at the electrode–electrolyte interface, Rsol is the solution resistance, Cde denotes the dielectric capacitance of the bulk electrolyte, and Zcell corresponds to the impedance contribution of the functionalized sensing interface. Rcell and Ccell represent the resistance and capacitance associated with the bioreceptor layer modeled in parallel. *Adapted from ref* ^101^. Copyright 2021 MDPI.

Upon immobilization of a stable antibody or antigen on an electroactive platform, the resulting construct, termed an immunobiosensor, enables the detection of a specific antigen or antibody through the formation of a stable immunocomplex with its corresponding receptor, generating a measurable signal via a transducer.^94^ In the most recent study on leprosy biosensors, Almeida et al.^95^ presented an electrochemical magneto-immunoassay integrated into a disposable microfluidic device. This assay used magnetic particles conjugated with a mimotopic peptide to capture *M. leprae*-specific antibodies and reported 91.2% sensitivity and 93.3% specificity with an area under the ROC curve (AUC) of 0.990. Importantly, it was also able to distinguish between PB and MB forms, with a sensitivity of 88.9% and specificity of 93.7%. In 2019, Yotsumoto Neto et al.^78^ introduced a photoelectrochemical immunosensor employing a recombinant mimetic peptide and CdS/Ni­(OH)_2_/FTO electrodes. The sensor responded strongly to leprosy-positive serum samples even at high dilutions (up to 1:10240), whereas negative samples diluted below 1:640 produced no significant photocurrent response. Unlike conventional electrochemical sensors that rely solely on redox reactions to generate current, photoelectrochemical (PEC) sensors exploit light to generate electron–hole pairs that enhance the redox reaction, enabling signal amplification and lower background noise.^96^

Among the different classes of biosensors, DNA-based biosensors have attracted significant attention due to their versatility and highly specific molecular recognition. These biosensors use DNA or DNA-derived structures (such as aptamers, DNAzymes, or DNA hairpins) as the recognition element to specifically detect nucleic acids, proteins, small molecules, or metal ions. The interaction between the target and the DNA probe generates a measurable signal, often via electrochemical, optical, or other transduction methods, enabling rapid, sensitive, and selective detection for applications in disease diagnostics, genetic analysis, and environmental monitoring.^97–99^ In 2021, Afonso et al.^100^ developed a DNA-based electrochemical biosensor consisting of graphite electrodes modified with poly­(4-aminophenol) and methylene blue as a hybridization indicator, achieving a detection limit of 1 × 10^–10^ mol/L with high selectivity for *M. leprae* DNA.

Optical biosensors detect biological interactions through light-based signals, providing high selectivity, rapid response, and remarkable sensitivity.^102^ They can operate via label-based or label-free approaches, the latter relying on phenomena such as evanescent fields, total internal reflection, ring resonance, or surface plasmon resonance (SPR).^103,104^ Among them, SPR stands out as a powerful technique that couples incident light and collective oscillations of free electrons (surface plasmons) at a metal–dielectric interface (Figure 4).^105^ This resonance is highly sensitive to changes in the local refractive index near the metal surface, which occur when biomolecules interact with a functionalized sensing layer.^83^ By detecting shifts in the resonance angle or wavelength, SPR enables real-time, label-free monitoring of biomolecular interactions, such as receptor–ligand binding, with high sensitivity.^106^

**Figure 4:**
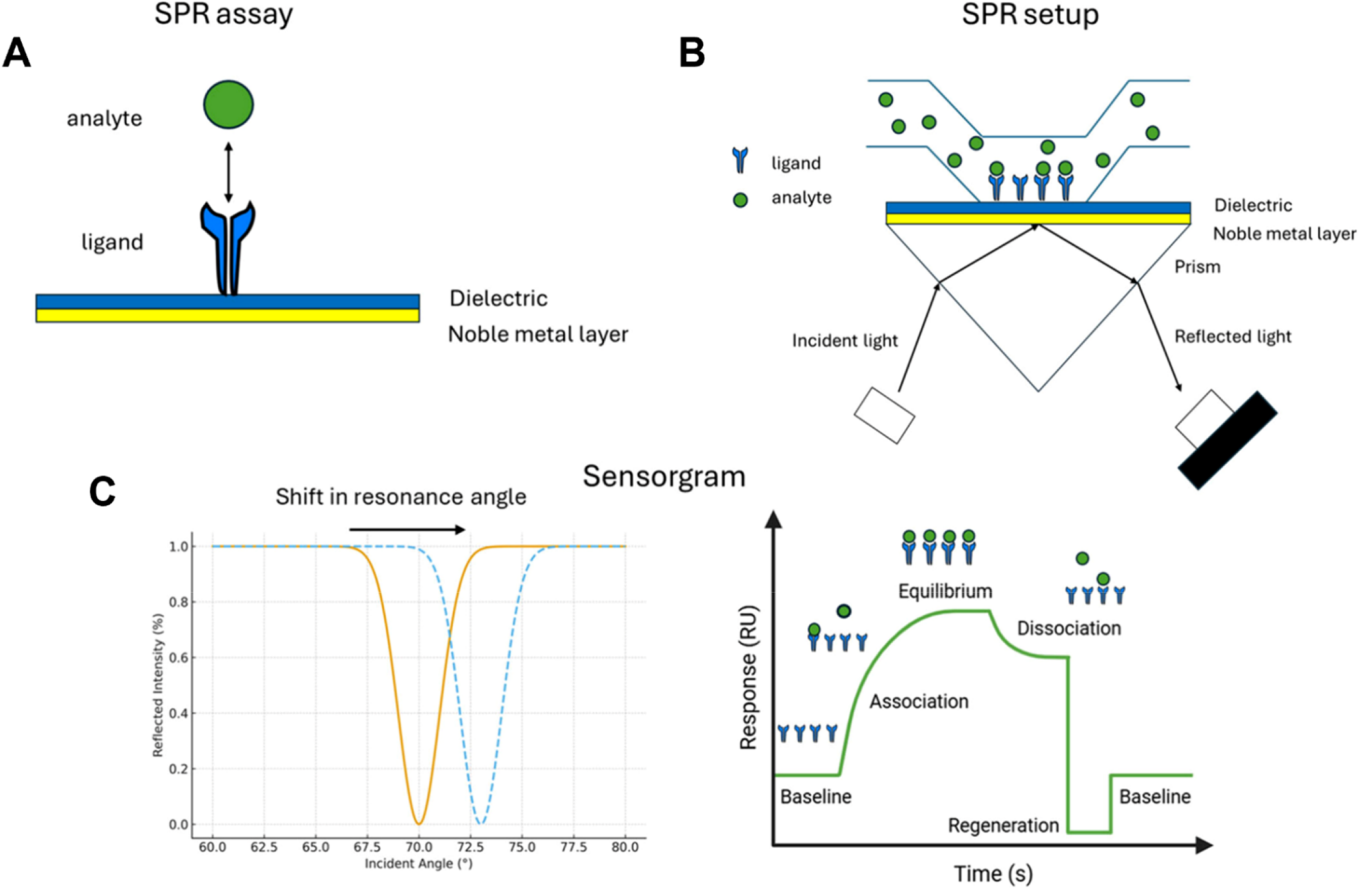
Schematic representation of the SPR setup. (A) In a standard SPR assay, one molecule, termed the ligand, is immobilized on the sensor surface. The sensor is prefunctionalized with specific surface chemistries to facilitate ligand attachment, enabling optimal interaction with its binding partner, the analyte. (B) A solution containing the analyte is then passed over the functionalized sensor surface, where ligand–analyte binding occurs through specific molecular recognition. (C) The minimum in the reflected light intensity shifts as the angle of incidence changes, corresponding to variations in the refractive index caused by mass accumulation on the sensor surface. This shift is recorded in a sensorgram that depicts the real-time association and dissociation kinetics of the analyte–ligand interaction as a function of time. *Reproduced from ref* ^108^. Copyright 2025 American Chemical Society.

In 2020, Lima et al.^107^ employed a PGL-I mimetic peptide (PGLI-M3) to evaluate an SPR assay using serum samples and compared the results with an ELISA performed with the same material. SPR was able to distinguish all forms of leprosy, including the detection of PB cases. The binding affinity of PGLI-M3 for serum antibodies was approximately 30-fold lower than that measured by ELISA. Only household contacts showed no significant angle variation in SPR measurements.

Another type of biosensor explored was the piezoelectric system, which is a mass-based biosensor, considered passive because it generates an electrical signal directly in response to external stimuli, without requiring additional energy sources.^109^ This is based on the piezoelectric effect, whereby mechanical stress applied to crystalline materials (e.g., quartz, Rochelle salts, lithium sulfate, ammonium phosphate, barium titanate, zirconate titanate, zinc oxide, aluminum nitride, and polyvinylidene fluoride) induces an electric potential across conductive plates. Conversely, applying an electric field may cause crystal deformation in its structure.^110^ The binding of biomolecules to the crystal surface increases the crystal surface mass, causing a shift in the resonance frequency (Figure 5). This frequency shift can be precisely measured and directly correlated with the amount of bound analyte.^111^ The capability of piezoelectric materials is attributed to their highly ordered atomic arrangement, which is characterized by a null net charge. When subjected to compressive or expansion forces in a particular direction, a charge imbalance occurs, leading to localized regions of differing charge density and the consequent generation of an electrical field.^112^

**Figure 5:**
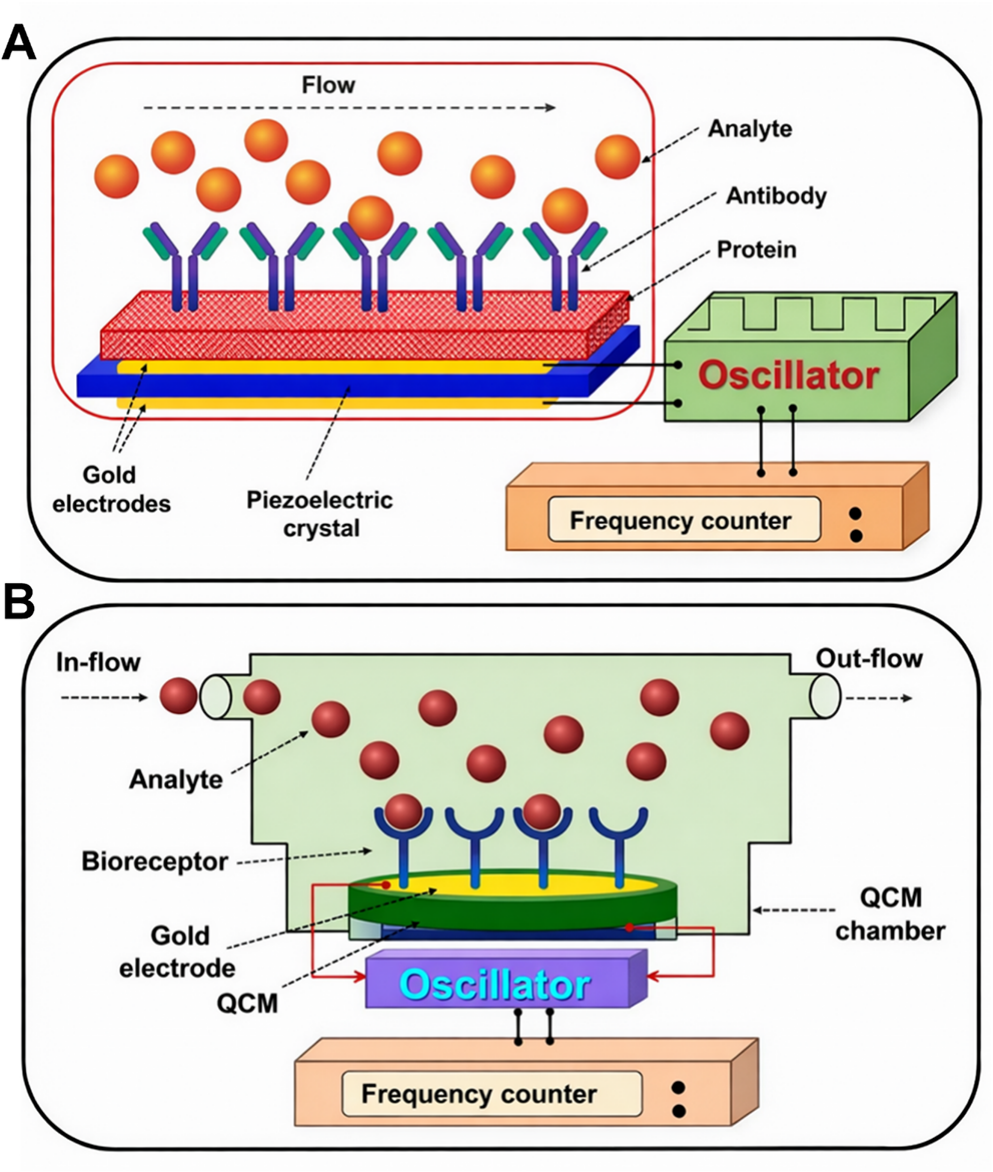
Schematic diagrams of mass-based biosensors. (A) Piezoelectric biosensor under flow conditions, where the binding of analyte to immobilized antibodies on a piezoelectric crystal leads to frequency changes measured by an oscillator and frequency counter. (B) Quartz crystal microbalance biosensor illustrating the QCM chamber, gold electrodes, surface-functionalized bioreceptors, and frequency response upon analyte interaction. *Adapted from ref* ^101^. Copyright 2021 MDPI.

Santana et al.^79^ developed a label-free piezoelectric immunosensor using a mimotope of the *M. leprae* Ag85B antigen integrated into a quartz crystal microbalance (QCM) platform. In this system, antibodies from leprosy-positive serum exhibited irreversible binding to the peptide-coated QCM surface with no desorption observed after washing. The device detected antibodies in leprosy patients, including those with paucibacillary forms, and diagnostic performance was further improved using a biotin–streptavidin ELISA assay, achieving detection in 91.7% of patients.

A biomimetic biosensor is a detection platform that mimics the structural and functional properties of biological systems such as enzymes, antibodies, or cells to achieve selective, stable, and scalable sensing. These sensors often use synthetic recognition elements, like molecularly imprinted polymers (MIPs) or bioinspired nanomaterials, integrated with transduction methods (electrochemical, optical, etc.).^83,113,114^ Kushwaha et al.^115^ reported a MIP-based sensor employing an electrochemical quartz crystal microbalance (EQCM) for detecting an *M. leprae* epitope, with a detection limit (LOD) of 0.161 nM, allowing pathogen detection even at very low blood concentrations, combining piezoelectric and electrochemical techniques.

Figure 6 depicts the different bioreceptors and respective analytes explored in leprosy biosensor research, and Table 2 provides a summary of the biosensors developed for leprosy diagnosis, as discussed in this review.

**Figure 6:**
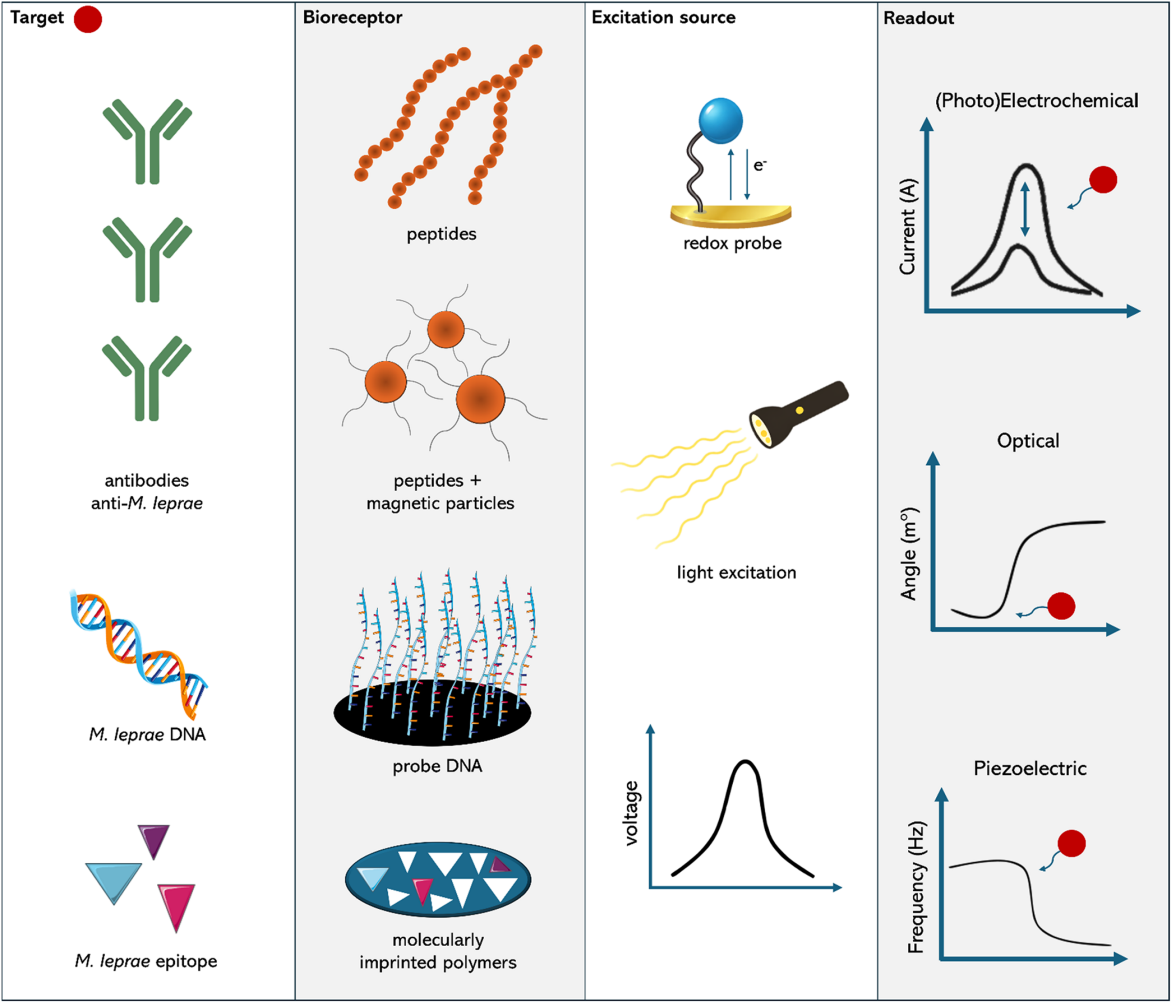
Overview of leprosy biosensors developed to date, highlighting their targets, bioreceptors, excitation sources, and readout strategies.

To date, thermal platforms represent a class of transducers that has not been investigated in the context of leprosy diagnostics. Thermal biosensors measure temperature changes generated by biological interactions, such as molecular or enzymatic reactions, and convert them into electronic signals proportional to the analyte concentration. While thermistor-based and calorimetric biosensors detect heat released or absorbed by biochemical reactions, the heat transfer method (HTM) monitors how binding of target molecules at a receptor layer alters heat transport across the solid–liquid interface, effectively changing the interfacial thermal resistance.^116–119^ This diversity of physical principles positions thermal platforms as a potential approach for the label-free detection of *M. leprae*-specific biomolecules.

Bioreceptor types that have not yet been explored in leprosy studies include cell-, phage-, and enzyme-based systems. Cell-based biosensors employ living cells as bioreceptors, providing physiologically relevant detection of analytes for applications such as drug screening, toxicity assessment, and disease studies,^120^ and could respond to pathogen-induced cellular changes, enabling functional readouts relevant for diagnosis. Phage-based biosensors use bacteriophages or their receptor-binding proteins (RBPs) as biorecognition elements for highly specific pathogen detection. They can be engineered with reporter genes and generate measurable signals through optical, electrochemical, or colorimetric transducers for rapid and sensitive readouts and could leverage *M. leprae*-specific receptor-binding proteins to detect bacterial antigens.^121,122^ Enzyme-based biosensors utilize enzymes as biorecognition elements, exploiting their catalytic specificity to detect target analytes. The resulting enzymatic reactions produce optical or electrochemical signals, enabling sensitive and selective detection in applications ranging from clinical diagnostics and food monitoring to environmental analysis,^123,124^ and might exploit mycobacterial enzymes or enzyme-linked amplification for signal enhancement.

### Current Challenges in Leprosy Biosensing

When compared with conventional diagnostic techniques, biosensors have shown competitive analytical performance. While SSS and histopathology provide high-to-moderate specificity (above 90 and 70%, respectively) and moderate sensitivity (30–78%),^36–38,44,45^ and molecular tests such as PCR typically reach approximately 75–80% sensitivity and 90–95% specificity,^32^ recent biosensor prototypes have reported sensitivities ranging from 88 to 91%, specificities above 90%, and very small limits of detection, such as 0.161 nM and 1 × 10^–10^ mol/L.^79,95,100,115^

Despite technological progress, several barriers must be overcome before biosensors can be widely adopted in clinical practice. One major challenge is the lack of large-scale multicenter validation studies that assess diagnostic performance across diverse populations, geographic settings, and clinical forms. Most leprosy biosensor studies to date have relied on small sample sizes under controlled laboratory conditions, which may not reflect the complexity of real-world scenarios.^78,79,95,100,115,125^ Another major limitation relates to PB patients, who present a lower bacterial load and weaker humoral immune responses. In such cases, biomarker concentrations in blood or other fluids may fall below biosensor detection thresholds, increasing the risk of false-negative results.^126^ Although established antigens such as PGL-I and LID-1 have proven useful, particularly for MB cases, they continue to show limited sensitivity for PB patients and asymptomatic infections. Therefore, the identification of novel and more sensitive antigens and other bioreceptors, especially through modern bioinformatics and epitope-mapping approaches, represents a critical step toward developing next-generation biosensors capable of diagnosing not only active disease but also latent infection and individuals at a high risk of progression.

Economic constraints also represent a substantial barrier. Although biosensors can achieve analytical performances similar to or exceeding those of traditional methods, there is a clear trade-off between sensitivity and cost. For example, microfluidic or nanostructured electrochemical devices may offer femtomolar-level detection but require specialized materials and fabrication steps that limit large-scale production.^61,127^ In contrast, paper-based or colorimetric lateral flow biosensors are low-cost and suitable for field use but can sacrifice sensitivity, particularly for PB cases. For instance, while the Brazilian public health system pays approximately US$4.80 for an immunochromatographic test, the cost of a single QCM crystal can reach around US$26.50.^15,128^ These costs, combined with cold-chain storage requirements for bioreagents, such as antibodies or enzymes, can hinder affordability and large-scale implementation in endemic regions.

Environmental factors also affect the biosensor performance. Variables such as temperature, humidity, pH, and ionic strength may compromise reagent stability, sensor surface activity, and signal transduction, particularly for electrochemical and photoelectrochemical mechanisms.^61,129–131^ It is known that environmental factors can cause signal variability exceeding 15–25% in electrochemical biosensors operated under noncontrolled temperature and humidity.^132^ This is particularly relevant for endemic tropical regions, where temperature fluctuations and high humidity can reduce sensor stability and reproducibility over time, especially in settings where laboratory infrastructure and environmental control are often limited.

In addition to technical issues, logistical and operational factors must be considered for the successful integration of biosensors into national leprosy control programs. These include training health workers, obtaining regulatory approvals, ensuring supply chain efficiency, and addressing user acceptability. Even a technically robust device may fail to improve case detection if it is not user-friendly or if the result interpretation is ambiguous. Designing biosensors with clear binary readouts and incorporating end-user feedback from the outset can enhance the adoption of decentralized health systems.

Despite these barriers, the technological advances reported over the past decade highlight the potential of biosensors to revolutionize leprosy diagnostics. However, translation into field-ready tools has so far remained limited, and to date, there is no evidence of these biosensors being introduced to the market. To examine the structure of scientific collaboration in this area, Figure 7 presents the authors and coauthors of the studies included in this review, resulting in maps of a collaboration network comprising 43 researchers. The tool and strategy used to construct these maps are described in the Supporting Information. In the co-authorship maps, clusters represent groups of collaborating authors, the node size reflects the number of publications, and edges indicate co-authorship links, with thicker lines corresponding to stronger collaborative interactions. The co-authorship structure revealed five main clusters (Figure 7A). The red cluster is the largest and includes authors such as Goulart, Brito-Madurro, and Afonso, showing connections to Yotsumoto-Neto, Lima, and others in the purple cluster. The blue cluster comprises an international group, including Singh, Ambareesh, Raghuwanshi, and Kushwaha. The green cluster represents the most internally connected group, with De Santana, De Moura, Chávez Olórtegui, and Alvarenga, which maintains several strong links to the yellow cluster, including authors such as Almeida and Faria.

A chronological visualization of collaborations from 2015 to 2025 highlights the evolution of the network over time (Figure 7B). Early publications (2015–2017, dark blue) consisted of small, internally connected groups, such as Singh, Kushwaha, Anand, and colleagues, with limited interaction across clusters. Between 2018 and 2020 (light blue to aqua), larger and denser groups emerged, such as those involving Sierakowski, De Santana, Fogaça and collaborators, indicating gradual expansion and initial integration between groups. From 2021 to 2023 (green), collaboration intensified, forming highly interconnected networks that bridged earlier clusters, with contributions from authors such as Goulart, Dias Oliveira, Araujo, and Lima. In the most recent period (2024–2025, yellow), new groups, including De Oliveira, Faria, and Fonseca, entered the network while retaining links with existing clusters, demonstrating continued growth and consolidation. Overall, the network evolved from isolated early groups into a dense and integrated collaborative structure over the past decade.

Methodological diversity is scientifically valuable and has yielded promising sensitivity and specificity data, particularly in some cases for PB forms. Ongoing collaborative frameworks that facilitate the exchange of design strategies, troubleshooting experiences, and, crucially, the sharing of well-characterized clinical samples and standardized protocols can enable cross-validation, accelerate the identification of optimal biomarker combinations, and streamline the translational pathway. This is exemplified by the use of the same PGL-I mimetic, PGLI-M3, by different groups employing diverse methodologies, which improved the detection of leprosy across different clinical forms and provided a version of a robust biomarker that could be more available in clinical practice.^79,107^

Moving forward, the integration of biosensors into public health frameworks will require technological validation, cost-effectiveness analyses, long-term reagent stability studies, and appropriate end-user training. Efforts should prioritize interlaboratory standardization and the creation of reference panels for sensitivity and specificity benchmarking, ensuring that new devices meet regulatory and operational requirements for use in endemic regions. Establishing coordinated research networks with open data sharing and interoperable methodologies is essential to accelerating the development of robust, affordable, and field-deployable point-of-care diagnostics for leprosy.

**Figure 7:**
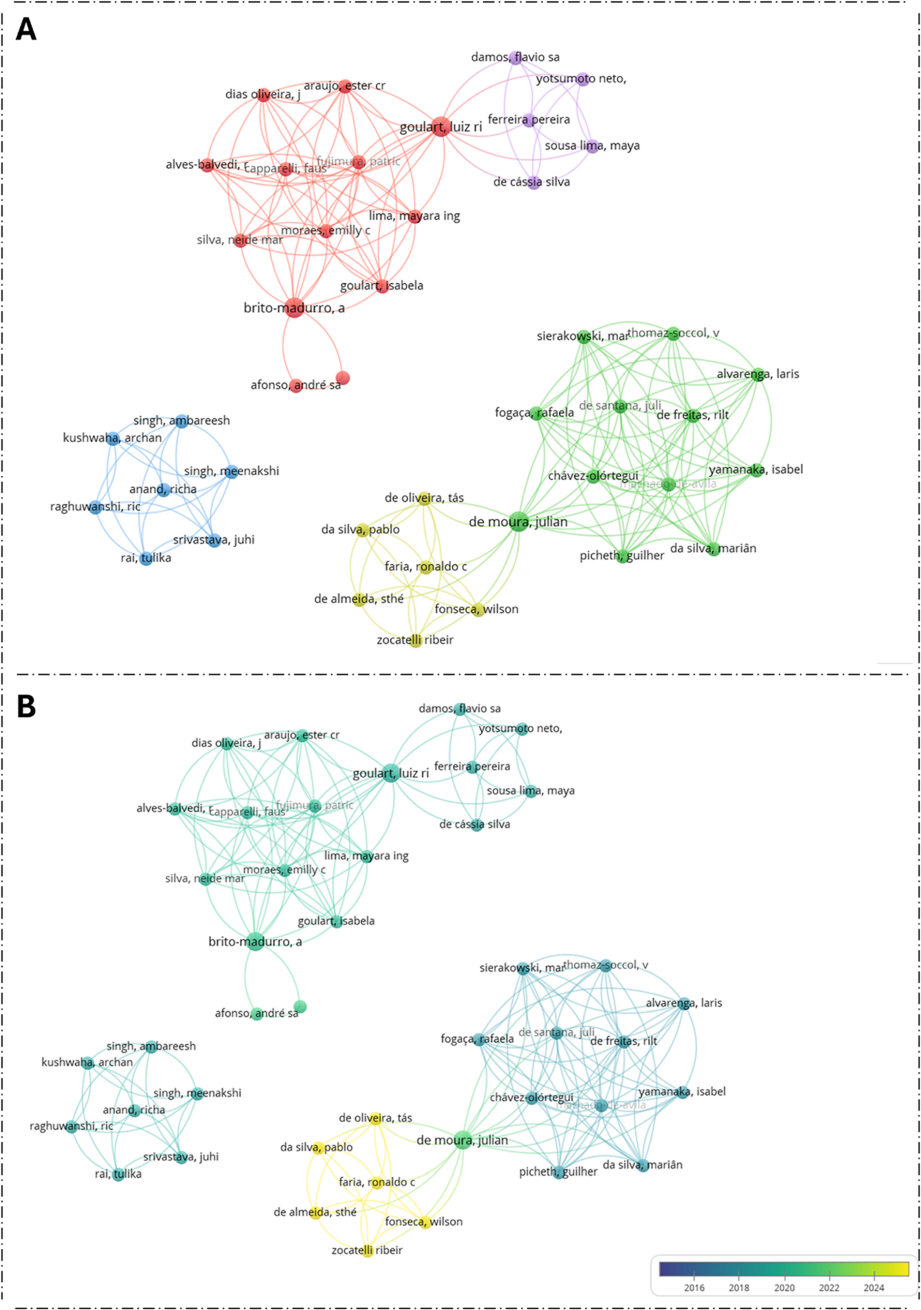
Co-authorship network of the 43 authors included in this review. (A) Network structure showing five main clusters identified by distinct colors. The node size represents the number of publications, and edges indicate co-authorship links, with thicker lines reflecting stronger collaborations. (B) Temporal evolution of the collaboration network from 2015 to 2025, with node colors indicating the year of publication (dark blue = 2015; yellow = 2025).

## Biosensors for Other Mycobacterial Infections

The challenges presented in the last section underscore the need to carefully evaluate and optimize biosensor platforms for leprosy diagnosis. Diverse detection strategies targeting other *Mycobacterium* species offer valuable insights that may be adapted for *M. leprae* diagnostics. The development of biosensors for the detection of other mycobacterial diseases has significantly advanced diagnostic capabilities, particularly in terms of speed, sensitivity, and specificity, especially in resource-limited regions.

For tuberculosis, such innovative technologies are crucial for the timely diagnosis and management of this most common mycobacterial disease in humans. Several studies have reviewed the development of multiple biosensors targeting different molecular markers, including DNA sequences, specific genes (e.g., IS6110, rpoB, and 16S rDNA), and mycobacterial antigens or proteins (e.g., ESAT-6, CFP10-ESAT6, MPT64, Ag85B, and HspX). These diagnostic tools employ techniques such as electrochemical, Förster resonance energy transfer (FRET), and surface plasmon resonance (SPR) often enhanced with nanocomposites gold nanoparticles (AuNPs), silver nanoparticles (AgNPs), carbon nanotubes (CNTs), graphene, metal–organic frameworks (MOFs), and magnetic nanoparticles, achieving detection limits ranging from femtomolar to nanomolar concentrations or as low as a few CFU/mL in serum, sputum, urine, and blood.^133–135^

Aptamer-based systems have also been developed by integrating nanohybrids, such as graphene oxide–metal–organic frameworks, to improve the electrochemical performance.^136^ Moreover, silicon nanowire field-effect transistors (SiNW-FET) have enabled rapid detection of mycobacterial proteins in sputum within minutes, without requiring pretreatment.^137^ Collectively, these advances demonstrate substantial progress in rapid, sensitive, and clinically applicable diagnostics for *Mycobacterium tuberculosis*. However, challenges remain in ensuring widespread accessibility and affordability.

For the *Mycobacterium avium* complex (MAC), biosensor platforms have also been explored. One example involved the development of an electrochemical DNA nanobiosensor for *M. avium* subsp. *paratuberculosis* (MAP) detection.^138^

In the case of *Mycobacterium kansasii*, loop-mediated isothermal amplification (LAMP) combined with lateral flow biosensors has been applied to detect species-specific rpoB gene sequences, allowing differentiation from other mycobacterial species.^139^

Overall, these biosensor platforms illustrate diverse detection strategies targeting different *Mycobacterium* species, each with distinct mechanisms and analytical capabilities. Importantly, many of these strategies can be adapted and optimized for *M. leprae* diagnostics, potentially accelerating the development of sensitive field-ready assays for leprosy.

## Future Perspectives and Research Directions

Significant opportunities remain to be explored in the application of biosensors for the detection of *M. leprae*. Technologies that have been extensively validated for other mycobacterial pathogens could be adapted and tested in the context of leprosy. Aptamer-based biosensors, for instance, remain an underexplored but highly promising path, while other transducer platforms, such as thermal and optical, could provide novel transduction strategies based on biologically driven heat-exchange mechanisms.

Investigating new molecular targets is also essential to improve the diagnostic sensitivity. Synthetic peptides and epitope-based antigens, identified through bioinformatics and immunoinformatics approaches, represent promising candidates for integration into biosensor platforms.^140^ Moreover, employing the same bioreceptors across different platforms could further enhance the detection accuracy, especially alternative forms of well-established bioreceptors, such as PGL-I. Another important research direction is the development of assays capable of detecting infection before symptom onset, thereby reducing transmission risk and preventing disease progression.

In this regard, fostering collaboration among research groups and strengthening cross-institutional networks will be critical. The sharing of well-characterized clinical samples, methodological frameworks, and validated target molecules can accelerate progress, promote cross-validation, and enhance reproducibility. This cooperative model may be decisive for translating biosensor prototypes into innovative, clinically relevant diagnostic tools for leprosy.

## Conclusions

The field of biosensors has evolved considerably, with substantial progress demonstrated across a range of infectious diseases. By integration of diverse detection strategies, such as electrochemical, optical, piezoelectric, and thermal platforms, often enhanced with nanomaterials, microfluidics, or innovative transduction mechanisms, biosensors have demonstrated their capacity to deliver rapid, sensitive, and clinically relevant results. These advances provide a strong foundation from which to extend biosensor applications to other neglected diseases, including leprosy.

In the context of leprosy, however, progress has been comparatively limited, with relatively few biosensor platforms developed to date despite the pressing clinical and epidemiological need. Current studies have highlighted the potential of biosensors to detect *M. leprae*-specific antibodies, DNA, and synthetic antigens detected with high accuracy, even in paucibacillary forms. The biosensors reviewed show distinct advantages, but all remain prototypes and, after assembly, require only direct sample application, except for the DNA-based sensor from Afonso et al., which requires prior extraction. Electrochemical immunosensors combined high accuracy with a portable format, making them the most suitable for point of care. Optical and photoelectrochemical platforms provide rapid detection and a wide dynamic range. Piezoelectric sensors, while highly sensitive, rely on more delicate, less integrated instrumentation, reducing portability compared to electrochemical devices. The DNA-based sensor achieves the lowest detection limit (1 × 10^–10^ mol/L) but requires a more complex sample preparation. Overall, electrochemical and photoelectrochemical sensors stand out for operational simplicity, while piezoelectric and DNA-based platforms offer higher analytical sensitivity at the cost of added complexity.

The future of leprosy diagnostic tools lies in the development of biosensors capable of detecting infection before symptom onset, enabling earlier treatment initiation and reducing transmission. Identifying novel biomarkers through bioinformatics-driven epitope mapping, incorporating aptamer-based recognition elements, and validating these approaches in multicenter large-scale studies represent decisive next steps. Furthermore, ensuring low-cost fabrication, environmental robustness, and user-friendly formats will be essential to achieving implementation in endemic, resource-limited settings.

Ultimately, biosensor technologies hold the potential not only to fill persistent gaps in leprosy diagnostics but also to transform surveillance and control strategies. Their successful translation from laboratory prototypes into field-ready devices will require multidisciplinary collaboration, standardized validation pipelines, and integration into national health programs. By a combination of scientific innovation with public health needs, biosensors may become a cornerstone in global efforts to achieve leprosy elimination.

## Supplementary Material



## Notes

Literature search methodology (PDF)

## Abbreviations

AUC: area under the curve
AgNPs: silver nanoparticles
AuNPs: gold nanoparticles
BI: bacillary index
CPE: constant phase element
CNTs: carbon nanotubes
DNA: deoxyribonucleic acid
EQCM: electrochemical quartz crystal microbalance
ELISA: enzyme-linked immunosorbent assay
FRET: Förster resonance energy transfer
FTO: fluorine-doped tin oxide
HTM: heat transfer method
IHP: inner helmholtz plane
LOD: limit of detection
MB: multibacillary
*M*. *leprae*: *Mycobacterium leprae*
MAC: *Mycobacterium avium* complex
MAP: *Mycobacterium avium* subsp. *paratuberculosis*
MOFs: metal–organic frameworks
mRNA: messenger RNA
NDO-HSA: natural disaccharide–human serum albumin
NDO-LID: natural disaccharide–LID
OHP: outer Helmholtz plane
PB: paucibacillary
PCR: polymerase chain reaction
PGL-I: phenolic glycolipid-I
Pra: *M. leprae* Pra gene
QCM: quartz crystal microbalance
RLEP: *M. leprae* repetitive element
rRNA: ribosomal RNA
SiNW-FET: silicon nanowire field-effect transistor
SSS: slit-skin smear
SPR: surface plasmon resonance
WHO: world health organization
