# Supplementary material for: Biosensor-Based Platforms for the Detection and Screening of Mycobacterium leprae Infection
Source: ACS Infect Dis. 2026 Jan 21;12(2):490–506. doi: 10.1021/acsinfecdis.5c00851 (PMC12910598; doi:10.1021/acsinfecdis.5c00851)
Supplement: Supplementary file 1 [file id5c00851_si_001.pdf]

Biosensor-based platforms for the detection and screening of *Mycobacterium leprae* infection

Augusto César Parreiras de Jesus[¥,μ], Ana Laura Grossi de Oliveira[μ], Flavia Di Scala[¥], Cristiane Alves da Silva Menezes[π], Lilian Lacerda Bueno[μ,#], Bart van Grinsven[¥], Rocio Arreguin-Campos[¥], Ricardo Toshio Fujiwara[μ,#], Thomas J. Cleij[¥]*

* augusto.parreirasdejesus@maastrichtuniversity.nl

[¥]Sensor Engineering Department, Faculty of Science and Engineering, Maastricht University, Duboisdomein 30, 6200MD, Maastricht, The Netherlands.

[μ]Post-Graduate Program in Infectious Diseases and Tropical Medicine, School of Medicine, Federal University of Minas Gerais, Av. Prof. Alfredo Balena 190, 30130-100, Belo Horizonte, Brazil.

[π]Department of Clinical and Toxicological Analysis, Faculty of Pharmacy, Federal University of Minas Gerais, Av. Pres. Antônio Carlos 6627, 31270-901, Belo Horizonte, Brazil.

[#]Department of Parasitology, Institute of Biological Sciences, Federal University of Minas Gerais, Av. Pres. Antônio Carlos 6627, 31270-901, Belo Horizonte, Brazil.

Supporting Information: Literature search methodology

Materials and Methods

For data collection, a comprehensive literature review was performed using a structured search strategy to retrieve academic papers published between 2015 and 2025 from the PubMed/MEDLINE, Scopus, and Web of Science databases, aiming to encompass a broad range of studies addressing the development of biosensor-based technologies for the diagnosis of mycobacterial infections, with a special focus on leprosy. The chosen timeframe reflects advances in the field over the last decade. A narrative synthesis of findings was compiled.

Search strategy

The search strategy combined controlled vocabulary and free-text terms applied to the Title, Abstract, and Keywords fields. Boolean operators were used to refine the query. The core terms included: “Biosensors” OR “Electrochemical sensors” OR “Immunosensor” OR “Photoelectrochemical immunosensors” OR “Electrochemical detection” OR “Recombinant proteins” OR “Synthetic peptides” AND “Mycobacterium” OR “Mycobacteria”. To ensure inclusiveness, equivalent terms in English, Spanish, and Portuguese were also incorporated. Descriptor terms were selected based on the most frequent and relevant keywords reported in reviews and primary research articles from the past ten years, ensuring coverage of both consolidated and emerging concepts related to biosensor-based diagnostic approaches for mycobacterial detection.

Study selection

All retrieved studies were uploaded into Rayyan (Qatar Computing Research Institute) for organization and processing. Duplicate records and articles that did not address the main objective of this chapter were excluded. In addition, manual searches were performed to identify relevant studies that might not have been captured through the electronic search. Titles and abstracts were independently screened for potential inclusion by two reviewers (ACPJ and ALGO). Any discrepancies were resolved through discussion with a third author (FS). Full-text screening was then conducted to include only studies that investigated the use of biosensor technologies for the diagnosis of mycobacterial infections. Finally, special attention was given to studies exploring the application of biosensors for the diagnosis of leprosy, specifically those focusing on the detection of *M. leprae* antigens.

Data collection and extraction, and result analysis

The following data were extracted from the full-text version of the included articles: first author, year of publication, biosensor type, *M. leprae* target antigen, detection platform, analytical sensitivity or detection limit, and key findings. Two reviewers independently extracted the data, and any discrepancies were resolved through discussion with a third author.

Zotero version 7.0.24 was used to export the references in .ris format, which were subsequently imported into VOSviewer version 1.6.20 (Centre for Science and Technology Studies, Leiden University, The Netherlands). This software was applied to analyze co-authorship networks and to generate visualization maps. Fig. 5 shows co-authorship maps, in which clusters represent groups of collaborating authors and node size indicates the number of publications. Co-authorship analysis was conducted by considering a minimum threshold of one publication per

author, resulting in the inclusion of 43 authors in the scientific collaboration network. This approach provided insights into collaborative patterns and the structure of research communities working on biosensor-based approaches for mycobacterial diagnosis.

Results

Identification of studies

The electronic search retrieved a total of 80 records (PubMed: n = 42, Scopus: n = 23, Web of Science: n = 15). After the removal of duplicates (n = 31), 49 unique studies remained for screening based on titles and abstracts. Of these, 15 were excluded because they did not investigate the use of biosensors for detecting mycobacterial infections in human diseases, focused on other non-mycobacterial conditions, or evaluated vaccine-related compounds. Subsequently, 34 full-text articles were assessed for eligibility. Among these, 28 studies reported the detection of *M. tuberculosis*, while 6 focused on the detection of *M. leprae* and were included in this chapter. The co-authorship network of the 43 authors presented in this chapter revealed five main clusters, each represented by a distinct color in the visualization map (Fig. 7). Node size corresponds to the number of publications per author, while the connections (edges) represent co-authorship links, with thicker lines indicating stronger collaboration.
